# Assessing the utility of an anti-malarial pharmacokinetic-pharmacodynamic model for aiding drug clinical development

**DOI:** 10.1186/1475-2875-11-303

**Published:** 2012-08-30

**Authors:** Sophie Zaloumis, Andrew Humberstone, Susan A Charman, Ric N Price, Joerg Moehrle, Javier Gamo-Benito, James McCaw, Kris M Jamsen, Katherine Smith, Julie A Simpson

**Affiliations:** 1 Centre for Molecular, Environmental, Genetic & Analytic Epidemiology, Melbourne School of Population Health, University of Melbourne, Level 3, 207 Bouverie St, Carlton, VIC 3053, Australia; 2 Medicines for Malaria Venture, Geneva, Switzerland; 3 Centre for Drug Candidate Optimisation, Monash University, Melbourne, Australia; 4 Global Health Division, Menzies School of Health Research, Charles Darwin University, Darwin, Australia; 5 Centre for Tropical Medicine, Nuffield Department of Clinical Medicine, University of Oxford, Oxford, United Kingdom; 6 Tres Cantos Medicines Development Campus, Tres Cantos, Spain; 7 Vaccine and Immunization Research Group, Murdoch Childrens Research Institute and Melbourne School of Population Health, University of Melbourne, Melbourne, Australia; 8 Bioinformatics Division, The Walter and Eliza Hall Institute of Medical Research, 1G Royal Parade, Parkville, VIC 3052, Australia; 9 Department of Medical Biology, University of Melbourne, Parkville, VIC, 3010, Australia

**Keywords:** Plasmodium falciparum, Pharmacokinetic-pharmacodynamic model, Anti-malarial combination therapy

## Abstract

**Background:**

Mechanistic within-host models relating blood anti-malarial drug concentrations with the parasite-time profile help in assessing dosing schedules and partner drugs for new anti-malarial treatments. A comprehensive simulation study to assess the utility of a stage-specific pharmacokinetic-pharmacodynamic (PK-PD) model for predicting within-host parasite response was performed.

**Methods:**

Three anti-malarial combination therapies were selected: artesunate-mefloquine, dihydroartemisinin-piperaquine, and artemether-lumefantrine. The PK-PD model included parameters to represent the concentration-time profiles of both drugs, the initial parasite burden and distribution across the parasite life cycle, and the parasite multiplication factor due to asexual reproduction. The model also included the maximal killing rate of each drug, and the blood drug concentration associated with half of that killing effect (*in vivo* EC50), derived from the *in vitro* IC50, the extent of binding to 0.5% Albumax present in the *in vitro* testing media, and the drugs plasma protein binding and whole blood to plasma partitioning ratio. All stochastic simulations were performed using a Latin-Hypercube-Sampling approach.

**Results:**

The simulations demonstrated that the proportion of patients cured was highly sensitive to the *in vivo* EC50 and the maximal killing rate of the partner drug co-administered with the artemisinin derivative. The *in vivo* EC50 values that corresponded to on average 95% of patients cured were much higher than the adjusted values derived from the *in vitro* IC50. The proportion clinically cured was not strongly influenced by changes in the parameters defining the age distribution of the initial parasite burden (mean age of 4 to 16 hours) and the parasite multiplication factor every life cycle (ranging from 8 to 12 fold/cycle). The median parasite clearance times, however, lengthened as the standard deviation of the initial parasite burden increased (i.e. the infection became more asynchronous).

**Conclusions:**

This simulation study demonstrates that the PD effect predicted from *in vitro* growth inhibition assays does not accord well with the PD effect of the anti-malarials observed within the patient. This simulation-based PK-PD modelling approach should not be considered as a replacement to conducting clinical trials but instead as a decision tool to improve the design of a clinical trial during drug development.

## Background

Despite significant progress in control of malaria over the last decade, it remains a major global health problem. Almost 40% of the world’s population live in malaria endemic areas, with each year about a quarter of a billion people experiencing clinical malaria and an estimated 655,000 malaria-related deaths [1]. With no vaccine currently available, malaria control relies on preventative measures (i.e. insecticidal bed nets and indoor residual spraying) and effective treatment with artemisinin-based combination therapy (ACT). ACT involves treatment with two or more anti-malarials; a fast acting but short lived artemisinin derivative and a less effective, but of longer duration, partner drug. ACT is recommended by WHO as the first-line treatment of uncomplicated falciparum malaria [2], but recent reports from western Cambodia raise concerns that *Plasmodium falciparum* has developed reduced susceptibility to oral artesunate [3,4]. In the context of emerging resistance to artesunate (the most widely used artemisinin derivative), it is critical that new anti-malarial treatments are developed and assessed.

Mechanistic within-host pharmacokinetic-pharmacodynamic (PK-PD) models that relate blood anti-malarial drug concentrations to the parasite-time profile have potential to aid anti-malarial drug development. Simulated parasite-time profiles for hypothetical patients can be generated from the mechanistic PK-PD model and incorporate between-patient variability in the drug concentration profiles. Comparisons of parasitological outcomes (e.g. distribution of parasite clearance times and proportion of patients cured) derived from these hypothetical individuals can then be used as a decision tool for assessing dosing schemes and potential partner drugs for new anti-malarial drugs. This simulation-based approach has been adopted previously using a within-host continuous-time PK-PD model for comparing dosing schemes of mefloquine [5,6], artesunate [7], chloroquine [8], and the ACT, mefloquine and artesunate [9]. More recently a parasite stage-specific discrete-time within-host PK-PD model has been developed, and a stochastic simulation-based approach implemented to compare dosing schemes for artesunate [10].

In this paper, the above stage-specific model was extended to account for the action of two or more anti-malarial treatments, and the anti-malarial pharmacodynamic parameters were determined by extrapolating from *in vitro* data. Using a Latin-Hypercube-Sampling approach [11], the sensitivity of the PK-PD model to particular parameters was assessed, by comparing, across different sets of parameter values, the parasitological outcomes derived from simulated parasite-time profiles of hypothetical patients.

## Methods

### Within-host pharmacokinetic-pharmacodynamic model

The within-host PK-PD model is based on that described in Saralamba *et al.*[10], which is a discrete-time model that incorporates the age distribution of the parasite population within the malaria patient. This model determines how the distribution of the parasite age changes post treatment as a consequence of the concentration of the anti-malarial drug. The general form of the discrete-time model (see Saralamba *et al.*[10] supplemental information for more detail) and PK-PD parameter values used to simulate individual parasitaemia-time profiles in the presence of anti-malarial combination therapies is described below.

Prior to drug administration, the initial parasite load of each patient (P_0*i*_) is distributed among the 48 hourly age intervals of the *P. falciparum* life cycle according to a Gaussian distribution with a mean age of *μ* hours and a standard deviation of *σ* hours (see Additional file 1).

The expected number of parasites in patient *i* ( $N_{i}\left(a,\;t\right)$) aged ‘*a*’ hours (where 1 ≤ *a* ≤ 48) at hourly time point *t* after drug administration, is expressed by the following difference equations,

(1) $$N_{i}\left(a,t\right)=N_{i}\left(a-1,\;t-1\right)e^{-k_{i}\left(a-1,t-1\right)}\;\text{for 1}<a\;\leq 48$$

and

(2) $$N_{i}\left(1,\;t\right)=PMF\times N_{i}\left(48,\;t-1\right)e^{-k_{i}\left(48,t-1\right)}\text{for }a=1\text{.}$$

In (1) and (2), $k_{i}\left(a,t\right)$ is the kill rate constant for the combination therapy of the parasites aged *a* hours at hourly time point t, and in (2), *PMF* is the parasite multiplication factor that represents the number of merozoites released per schizont at the end of the 48 hour life cycle that successfully reinvade red blood cells.

The kill rate constant for a particular combination therapy was calculated as follows:

(3) $$k_{i}\left(a,t\right)=k_{drug_{1},i}\left(a,t\right)+k_{drug_{2},i}\left(a,t\right)$$

where $drug_{\text{j}}$, for j = 1, 2, denotes one of the drugs comprising a combination therapy. The kill rate constant in (3) assumes that the effects of $drug_{1}$ and $drug_{2}$ on the parasite population *in vivo* are independent of one another.

The relationship between the kill rate constant for each drug and the drug concentration is given by:

(4) $$k_{i}\left(a,t\right)=k_{\text{max}}\left(a\right)\times \left[c_{i}{\left(t\right)}^{\gamma }/\left(c_{i}{\left(t\right)}^{\gamma }+EC_{50}^{\gamma }\right)\right]$$

where $c_{i}\left(t\right)$ is the plasma drug concentration at time *t* for patient *i*, *γ* is the slope of the concentration-effect relationship, $EC_{50}$ is the blood drug concentration *in vivo* that gives 50% parasite killing and *k*_max_ is the maximal killing rate of each drug (which was assumed to be constant across the parasite ages ‘*a*’ where the drug is known to have an effect).

The drug concentration-time curve $c_{i}\left(t\right)$ for each drug assumes the form of a structural PK model, for example a one- or two- compartment PK model with first-order absorption and elimination from the central body compartment.

### Simulation study

The model was implemented using R [12]. Parasite counts at several different time points and in the presence of anti-malarial combination therapies were simulated from this discrete-time model for hypothetical malaria patients. The summary measures that were derived from each simulated parasite count-time curve were: (i) the hypothetical patient was clinically cured (defined as the parasite count falling below 2.5 × 10^8^ parasites (i.e. 50 parasites per μL) and not reappearing above 2.5 × 10^8^ parasites by day 63 of follow-up); and (ii) the parasite clearance time (PCT) (hours), defined as the time for the circulating parasite count (parasites aged approximately 1 to 26 hours) to decrease below 2.5 × 10^8^ parasites. Circulating parasite counts were calculated from the total parasite counts following Saralamba *et al.*[10], where sequestration was estimated to start at a parasite age of 11 hours and the number of parasites older than 11 hours circulating in the blood was assumed to decrease exponentially.

The anti-malarial combination therapies selected for this simulation study were three artemisinin-based combination therapies: artesunate-mefloquine, dihydroartemisinin-piperaquine, and artemether-lumefantrine. Studies of *in vitro* interactions between the pharmacodynamic effects of the drugs have shown no interaction between dihydroartemisinin and piperaquine [13], and a small amount of synergy between artesunate and mefloquine [14], and between artemether and lumefantrine [15]. Thus, the assumption of independent pharmacodynamic effects of each drug in the combination therapies selected for this simulation study seems reasonable, especially considering the short amount of time (approximately six hours for dihydroartemisinin and twelve hours for artemether) that the drug concentrations of the artemisinin derivatives are present in the blood.

### Pharmacokinetic-pharmacodynamic parameters

In order to assess the utility of the PK-PD model for determining patient outcomes (described above) the sensitivity of the model to parameter values was explored. This was implemented using Latin-Hypercube-Sampling (LHS). LHS is a method which is used to randomly sample over large parameter spaces in an evenly distributed manner [11].

Before carrying out the LHS sampling, pharmacokinetic profiles of each anti-malarial drug for the three artemisinin-based combination therapies were simulated for 100 hypothetical patients. The dosing regimen used was the regimen recommended by the WHO ( [2]; see Table 1) and the PK profiles were simulated using parameter values and between-subject variability obtained from the literature (see Table 2 and Additional file 2) [16-19].

**Table 1:** Dosing regimen for each artemisinin-based combination therapy (ACT)

**ACT**	**Dosing regimen (WHO)**
ARS-MQ	ARS 4.0 mg/kg and MQ 8.3 mg/kg at 0, 24, 48 h
ART-LF	ART 80.0 mg/kg and LF 480.0 mg/kg at 0, 8, 24, 36, 48, 60 h
DHA-PQ	DHA 4.0 mg/kg and PQ 18.0 mg/kg at 0, 24, 48 h

ARS – artesunate, ART – artemether, DHA – dihydroartemisinin, LF – lumefantrine, MQ – mefloquine, PQ – piperaquine.

**Table 2:** **Population pharmacokinetic parameter values (BSV%)**^**†**^**for each drug**

	**Drug**				
**PK**^**a**^**parameter**	**ARS/DHA**^**b**^	**ART**	**LF**	**MQ**	**PQ**^**c**^
k_a_ (/h)	19.7^d^	0.37	0.17	7^d^	0.717
	(26.5%)	(63%)	(52%)	(26%)	(168%)
CL/F (L/h)	24.2^e^	180	7.04	0.8^e^	66
	(22.4%)	(50%)	(16%)	(33%)	(42%)
V/F (L)	0.83^f^	217	-	10.2^f^	-
	(50%)	(30–50%)		(51%)	
V_c_/F (L)	-	-	103	-	8660
			(30–50%)		(101%)
Q/F (L/h)	-	-	4.08	-	131
			(30–50%)		(85%)
V_p_/F (L)	-	-	272	-	24000
			(30–50%)		(50%)

ARS – artesunate, ART – artemether, DHA – dihydroartemisinin, LF – lumefantrine, MQ – mefloquine, PQ – piperaquine.

^†^Parameter values were taken from the literature: dihydroartemisinin [16], artemether [17], lumefantrine [17], mefloquine [18], piperaquine [19]. Between-subject variability (BSV%) presented as standard deviation multiplied by 100 (lognormal error model).

^a^k_a_ – absorption rate constant; CL/F – clearance; V/F – volume of distribution; Vc – volume of central compartment; Q – inter-compartmental clearance; and Vp –volume of peripheral compartment.

^b^Dihydroartemisinin profiles were simulated for artesunate, since dihydroartemisinin is the primary active metabolite of artesunate and artesunate is considered the pro-drug.

^c^PK parameters are for a 48kg individual.

^d^/day.

^e^L/kg/day.

^f^L/kg.

The PD parameters were varied across the LHS experiment to capture both biological and empirical uncertainty. The definition of each PD parameter and the statistical distribution selected for each PD parameter are given in Table 3 and Table 4, respectively. For the parameters that are drug independent, the distributions for the parameters which determine how the parasites are distributed across the 48 hours of the parasite life cycle (*μ* and *σ*) were sourced from PK-PD modelling of uncomplicated falciparum malaria patients [10] and the distribution for the parasite multiplication factor (PMF) was obtained from modelling of data collected from syphilis patients treated with an induced malaria infection [20,21].

**Table 3:** Parameter definitions for the within-host pharmacokinetic-pharmacodynamic model

**Parameter**	**Description**
*μ*_*IPL*_	Mean of the age distribution of the initial parasite burden
*σ*_*IPL*_	Standard deviation of the age distribution of the initial parasite burden
*PMF*	Parasite multiplication factor (/48 h cycle)
*k*_*max*_	Maximal killing rate of the drug / h
*γ*	Slope of *in vivo* concentration-effect curve
*EC*_50_	*In vivo* concentration when killing rate is 50% of the maximum

**Table 4:** Statistical distributions selected for each pharmacodynamic parameter

**Parameter**	**Drug**	**Distribution**^**b**^	**Additional details**
***μ***_***IPL***_^a^	*DU*(4, 16)		
***σ***_***IPL***_^a^	*DU*(2, 8)		
***PMF***^a^	*TRI*(8, 12, 10)		
***k***_**max**_	ARS/DHA	*TRI*(0.26, 0.47, 0.37)^c^	*PRR* = 10^5.28^; *KZ* = 38
	ART	*TRI*(0.12, 0.33, 0.22)^c^	*PRR* = 10^2.9^; *KZ* = 38
	LF	*TRI*(0.18, 0.54, 0.36)^c^	*PRR* = 10^3^; *KZ* = 22
	MQ	*TRI*(0.11, 0.46, 0.28)^c^	*PRR* = 10^2.25^; *KZ* = 22
	PQ	*TRI*(0.33, 0.65, 0.49)^c^	*PRR* = 10^4.6^; *KZ* = 24
*γ*	ARS/DHA	ln*N*(1.31, 0.65)	
	ART	ln*N*(1.53, 0.31)	
	LF	ln*N* (0.81, 0.58)	
	MQ	ln*N* (0.97, 0.54)	
	PQ	ln*N* (1.35, 0.66)	
*EC*_50_ (ng/mL)	ARS/DHA	*U*(1.44, 532.05)	*a* = $IC_{50}$ (adjusted)(ng/mL)^b,d^; *b* = 0.5×C_max_ (ng/mL)^b,d^
	ART	*U*(4.38, 46.20)	
	LF	*U*(1.75, 2331.60)	
	MQ	*U*(20.48, 1087.22)	
	PQ	*U*(11.56, 94.19)	

ARS – artesunate, ART – artemether, DHA – dihydroartemisinin, LF – lumefantrine, MQ – mefloquine, PQ – piperaquine.

^a^Drug independent parameters.

^b^DU – Discrete-uniform (*DU (a, b)*; *a* <*b*; *a* and *b* positive integers); TRI – Triangular (*TRI* (*a, b, c*); *a* <*c* <*b*; *a*, *b* and *c* real numbers); *lnN* – Log-normal (ln*N* (*μ* _ln_, *σ* _ln_); *μ* _ln_ and *σ* _ln_ positive real numbers); and U – Continuous-uniform: (*U* (*a*, *b*); *a* <*b*; *a* and *b* real numbers).

^c^The mode of the triangular distribution for $k_{\max}$ (*c*) was calculated from the following expression: $k_{\max}=\left[\left(1/KZ\right)\times \ln\left(PRR\right)\right]+\left[\left(1/48\right)\times \ln\left(PMF\right)\right]$, where *PRR* is the parasite reduction ratio for the drug in the corresponding row; *KZ* is the length of the killing zone in hours for each drug in the corresponding row; *PMF* in this expression equals 10 parasites / 48 h. The lower /upper limit of the triangular distribution for $k_{\max}$ (*a/b*) is calculated by evaluating the latter expression $k_{\max}$ at a *PRR* decreased/increased by 50-fold (*KZ* remains unchanged).

^d^$IC_{50}$ (adjusted) = $IC_{50}$ × (100 – BM / 100) × (100 / 100 - HPPB) × HWB;

BM – binding media; HPPB – human plasma protein binding; HWB – human whole blood to plasma ratio. IC50 (adjusted) values for each drug are given in Table 5 and C_max_ is the peak plasma concentration of a drug after administration.

The maximal killing rate (k_max_) of the drug was assumed to follow a triangular distribution. The middle values were taken from published clinical data [3,22,23]. Piperaquine was the only drug with no clinical studies of it administered as a monotherapy and the mode was set to a value equal to that derived from *in vitro* experiments [24]. The minimum and maximum values of the triangular distribution for each anti-malarial correspond to a 50-fold decrease and 50-fold increase in the number of parasites killed every 48 hours. Artesunate, dihydroartemisnin and artemether were assumed to kill parasites aged 6 to 44 hours; mefloquine 18 to 40 hours; piperaquine (assumed similar to chloroquine) 12 to 36 hours; and lumefantrine (assumed similar to mefloquine) 18 to 40 hours [25].

The slope of the concentration-effect curve (i.e*. in vivoγ*) was assumed to have the same statistical distribution (i.e. Log-normal) and parameter values (mean and standard deviation on log_e_ scale) as the *in vitroγ* which was derived from modelling of *in vitro* concentration-effect curves measured from a large number of parasite isolates.

The distribution of the EC50 for each anti-malarial drug is unknown, therefore, the conservative continuous-uniform distribution was chosen with the minimum value set to the adjusted *in vitro* IC50 and the maximum value equal to half of the maximum concentration of the population mean PK profile.

Five thousand parameter sets were selected from the above statistical distributions using LHS. For each parameter set, simulated parasite count versus time profiles for the 100 hypothetical patients (with the PK profiles determined above) were derived for each artemisinin-based combination therapy. The initial parasite burden for each of the 100 hypothetical patients was randomly selected from a log-normal distribution with a geometric mean of 1.14 × 10^11^ (i.e. parasitaemia of 22746 parasites per μL) and a standard deviation on the log-scale of 1.13. The initial parasite burdens for the 100 patients did not vary with LHS parameter set or with the artemisinin combination therapy used in the simulation.

### Determination of the *in vitro* IC50 and slope (γ) of concentration-effect relationship

Estimates of the *in vitro* IC50 (not corrected for binding) and *γ* for artesunate, dihydroartemisinin, mefloquine, piperaquine, and lumefantrine (refer to Table 5), were determined from statistical modelling of individual isolate effect versus drug concentration curves. The fresh *P. falciparum* parasite isolates were obtained from blood samples of 487 patients attending outpatient clinics in Papua, Indonesia between 2004 and 2010 [26]. The *in vitro* drug susceptibility was determined using the World Health Organization guidelines for schizont maturation tests. The *in vitro* data for artemether were measured at the Swiss Tropical and Public Health Institute (Switzerland; Basel) against asynchronous intraerythrocytic forms of the *P. falciparum* strain NF54 (obtained from MR4) using the [3H]-hypoxanthine incorporation assay [27].

**Table 5:** **Parameter values derived from *in vitro *experiments**

**Parameter**	**ARS/DHA**	**ART**	**LF**	**MQ**	**PQ**
IC50 measured concentration(nM)^†^	1.39	-	13.42	9.34	17.67
Molecular weight (g/mol)	384.4	-	528.9	378.3	535.5
IC50 concentration(ng/mL)	0.53	2.64	7.1	3.53	9.46
Binding to media of *in vitro* experiment (0.5% Albumax) (% bound)	82.8	82.8	99.6	85	98.1
Human plasma protein binding (% bound)	93	91.7	98.7	95.6	98.6
Human whole blood to plasma ratio	1.1	0.8	0.8	1.7	0.9
Scalar (adjusted)	2.7	1.66	0.25	5.8	1.22
IC50 (adjusted) concentration (ng/mL)^†^	1.44	4.38	1.75	20.48	11.56
*γ* (Slope of concentration-effect curve)	3.72	4.61	2.24	2.63	3.48
SD of *γ* on log-scale	0.65	0.31	0.58	0.54	0.66

ARS – artesunate, ART – artemether, DHA – dihydroartemisinin, MQ – mefloquine, PQ – piperaquine, LF – lumefantrine.

^†^Geometric mean.

The *in vitro* free drug IC50 was calculated from the measured *in vitro* IC50 (uncorrected for binding) by multiplying by the unbound fraction in the *in vitro* testing media. This value was then converted to an adjusted *in vitro* IC50 in whole blood (to represent concentrations comparable to an *in vivo* situation) by first dividing by the free fraction in plasma and then multiplying by the whole blood to plasma ratio (see Table 5).

Estimates of drug binding to the *in vitro* testing media (i.e. 0.5% Albumax in RPMI) and human plasma were determined using ultracentrifugation. Briefly, blank Albumax media and plasma were each spiked with compound and divided into six aliquots; three aliquots were subjected to ultracentrifugation (Beckman Optima XL-100K Ultracentrifuge, Rotor type 42.2 Ti; 223,000 x g) for 4.2 hours at 37°C to pellet the proteins whereas the remaining three aliquots served as controls and were incubated at 37°C for the same time period but without centrifugation. Controls were also stored at −20°C to confirm sample stability over the centrifugation period. Aliquots of the supernatants from the ultracentrifuged samples were first diluted 1:1 in acetonitrile, assayed by LC-MS and the responses compared to a calibration curve prepared in 50% aqueous acetonitrile to determine the unbound (i.e. free) concentration. Control concentrations in each matrix were determined using LC-MS by first precipitating the proteins with acetonitrile (3:1 acetonitrile: matrix) and then comparing the responses to a calibration curve from a blank matrix prepared using the same protein precipitation procedure. The free fraction in each matrix was then determined from the ratio of the average unbound (e.g. free) concentration to the average total concentration in each matrix.

The whole blood to plasma partitioning ratio (B/P) was determined by spiking aliquots of whole blood or plasma maintained at 37°C with compound, incubating for 2 min, and then centrifuging the whole blood sample to obtain the plasma fraction. Both the plasma fraction of whole blood and the plasma control were assayed for compound by LC-MS as described above. The blood to plasma ratio was calculated from the ratio of the concentration in the plasma control (used as a surrogate for the total whole blood concentration since whole blood assays were not available for each compound) to that in the plasma fraction of whole blood. The two-minute time point was used to avoid confounding issues due to potential blood instability; rapid equilibration between plasma and erythrocytes was assumed.

## Results

### Sensitivity of pharmacokinetic-pharmacodynamic (PK-PD) model to parameter values

**Figure 1:**
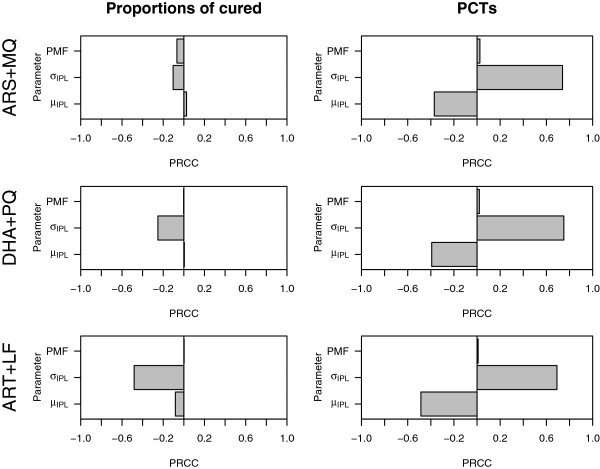
**Tornado plots of partial rank correlation coefficients, indicating the importance of each drug independent parameter’s (mean and standard deviation of the age distribution of the initial parasite burden, i.e. *μ *and *σ*, and the PMF) uncertainty in contributing to the variability in the proportion cured (left) and parasite clearance time (PCT) (right) for each artemisinin combination therapy.**

Examined first was the model’s sensitivity to the three parameters that describe the function of the parasite: the parasite multiplication factor (PMF), the mean for the age distribution of the initial parasite burden (*μ*), and the standard deviation of the age distribution of parasites within each host (*σ*). For each drug combination therapy and model output (proportion clinically cured and PCT), Figure 1 shows tornado plots of the partial rank correlation coefficients (PRCCs). The magnitude of the PRCC indicates the importance of the uncertainty in estimating the parameters governing the age distribution of the initial parasite burden (*μ* and *σ*) and the PMF in contributing to the variability in the proportion clinically cured and the PCTs. A negative PRCC indicates that the model output tends to decrease as the parameter increases and a positive PRCC indicates that the model output tends to increase as the parameter increases.

Figure 1 (left panel) shows that the proportion clinically cured (for ease of exposition referred to subsequently as proportion cured) after treatment with artemether-lumefantrine was sensitive to changes in the standard deviation of the age distribution of the initial parasite burden, and that it tended to decrease as the standard deviation of the age distribution (i.e.*σ*) increases (i.e. as the infection becomes more asynchronous). The proportion cured after treatment with artemisinin-lumefantrine was not strongly influenced by changes in the mean of the age distribution of the initial parasite burden (i.e. *μ*) and the PMF. The proportion cured for the remaining artemisinin combination therapies was not very sensitive to changes in the parameters defining the age distribution of the initial parasite burden (i.e. *μ* and *σ*) and the PMF.

The PCTs for all three artemisinin combination therapies were sensitive to changes in the mean and standard deviation of the age distribution of the initial parasite burden (see Figure 1, right panel). The PRCCs plotted in the right panel of Figure 1 show that the PCTs for all three artemisinin combination therapies tended to lengthen as the standard deviation of the age distribution increased (or the infection became more asynchronous) and tended to shorten as the mean of the age distribution increased.

Tornado plots of the PRCCs between the model outputs and the drug dependent parameters (EC50, k_max_ and *γ*) for each artemisinin combination therapy are also provided in Additional file 3.

#### Artesunate-mefloquine

**Figure 2:**
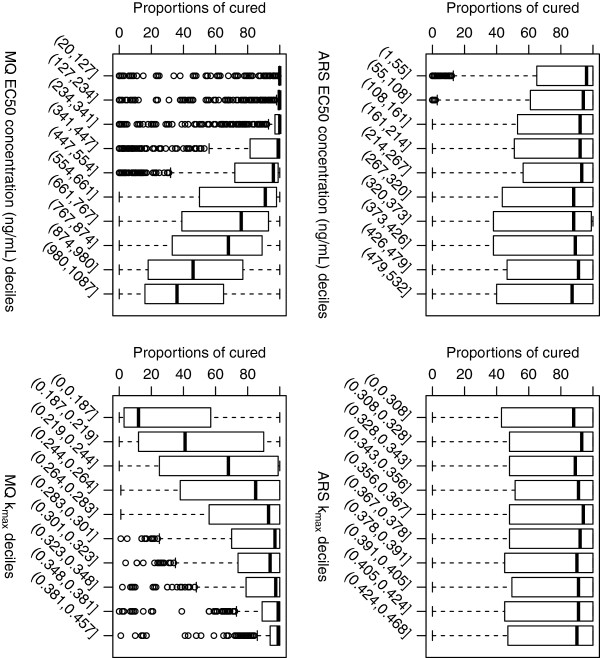
**Distribution of proportion cured within the EC50 and k**_**max**_**deciles derived from the 5000 parameter sets for the anti-malarial combination therapy, artesunate (ARS) and mefloquine (MQ).** Top panels are for artesunate (EC50 – left hand side, k_max_ right hand side) and bottom panels are for mefloquine (EC50 – left hand side, k_max_ right hand side). Each individual box (with whiskers) represents the distribution of the proportion cured simulated for 5000 parameter values of EC50 (or k_max_) within the range of cut-off values of that decile grouping, all other parameters were varied.

**Figure 3:**
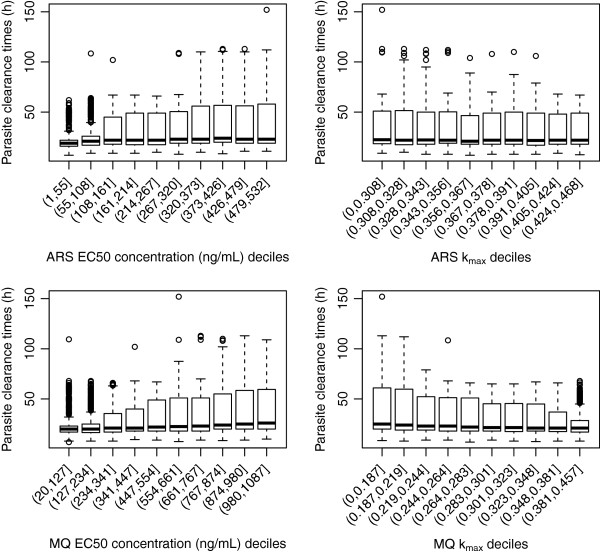
**Distribution of parasite clearance times (hours) within the EC50 and k**_**max**_**deciles derived from the 5000 parameter sets for the anti-malarial combination therapy, artesunate (ARS) and mefloquine (MQ).** Top panels are for artesunate (EC50 – left hand side, k_max_ right hand side) and bottom panels are for mefloquine (EC50 – left hand side, k_max_ right hand side).

Figure 2 depicts the distribution of the proportion cured for the combination therapy artesunate-mefloquine across deciles of the 5,000 parameter values for EC50 (drug concentration *in vivo* that corresponds to 50% parasite killing) and k_max_ (maximum killing rate constant) for both artesunate and mefloquine. The proportion of patients cured from the simulated parasite-time profiles was highly correlated with the *in vivo* EC50 and k_max_ for mefloquine, whereas the influence of the artesunate parameter values was marginal. Lower killing rates of mefloquine predicted a reduction in the percentage of patients being cured, the median value ranging from 10% to 95% for k_max_<0.187 (i.e. Parasite Reduction Ratio (PRR) <10^1.33^) up to k_max_>0.301 (i.e. PRR>10^2.42^). Only when the EC50 concentrations of mefloquine were between 447 to 554 ng/ml, much higher than the adjusted *in vitro* IC50 value (21 ng/ml), was the predicted median proportion cured similar to that observed in clinical studies [3,28,29]. With EC50 values between 661–874 ng/ml approximately 75% (on average) of the hypothetical patients were predicted to be cured. Figure 3 illustrates that PCTs are not vulnerable to the EC50 and k_max_ values of mefloquine and artesunate. Furthermore the values of the slope of either the artesunate or mefloquine concentration-effect curves had no association with any of the model outputs (see Additional files 4A-B).

#### Dihydroartemisinin-piperaquine

**Figure 4:**
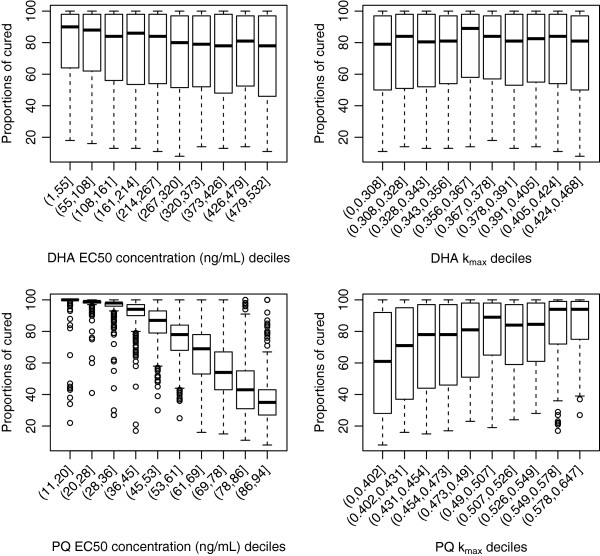
**Distribution of proportion cured within the EC50 and k**_**1**_**deciles derived from the 5000 parameter sets for the anti-malarial combination therapy, dihydroartemisinin (DHA) and piperaquine (PQ).** Top panels are for dihydroartemisinin (EC50 – left hand side, k_max_ right hand side) and bottom panels are for piperaquine (EC50 – left hand side, k_max_ right hand side).

**Figure 5:**
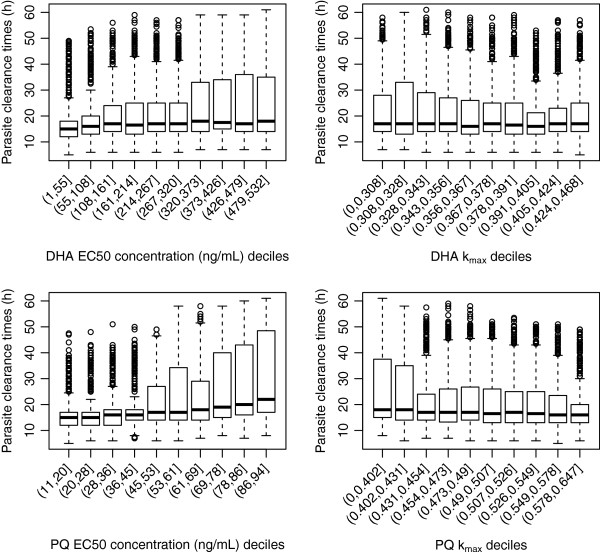
**Distribution of parasite clearance times (hours) within the EC50 and k**_**1**_**deciles derived from the 5000 parameter sets for the anti-malarial combination therapy, dihydroartemisinin (DHA) and piperaquine (PQ).** Top panels are for dihydroartemisinin (EC50 – left hand side, k_max_ right hand side) and bottom panels are for piperaquine (EC50 – left hand side, k_max_ right hand side).

Similarly for the combination of dihydroartemisinin-piperaquine, the proportion of patients cured correlated closely with the *in vivo* EC50 for the long acting partner drug, piperaquine (Figure 4), but was not influenced by values of EC50 or k_max_ of dihydroartemisinin. However in contrast to the mefloquine containing combination, values of piperaquine k_max_ were only weakly associated with the proportion cured. The median proportion cured changed dramatically from 100% to 40% across the deciles of EC50 values for piperaquine, with EC50 values of 45 to 53 ng/ml corresponding to a median of 95%. Figure 5 highlights that PCTs are not sensitive to k_max_ values of dihydroartemisinin and piperaquine but are marginally sensitive to the EC50 values of piperaquine. The values of the slope of either the dihydroartemisinin or piperaquine concentration-effect curves had no associations with the model outputs (see Additional files 5A-B).

#### Artemether-lumefantrine

**Figure 6:**
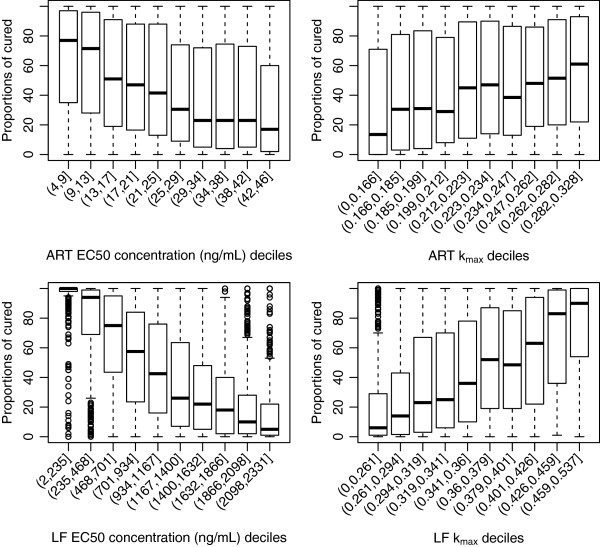
**Distribution of proportion cured within the EC50 and k**_**1**_**deciles derived from the 5000 parameter sets for the anti-malarial combination therapy, artemether (ART) and lumefantrine (LM).** Top panels are for artemether (EC50 – left hand side, k_max_ right hand side) and bottom panels are for lumefantrine (EC50 – left hand side, k_max_ right hand side).

**Figure 7:**
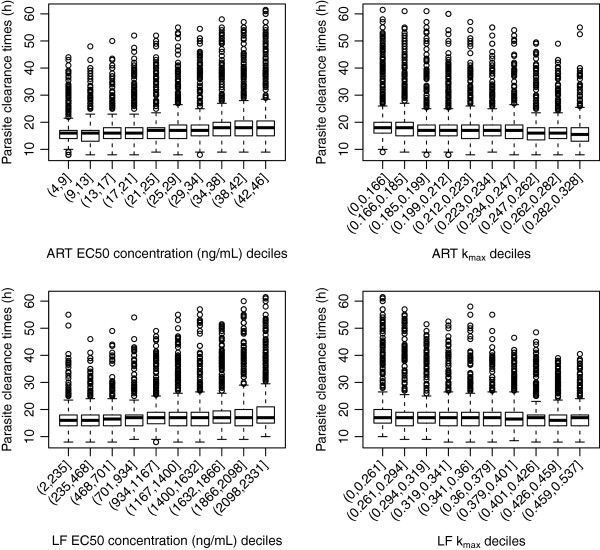
**Distribution of parasite clearance times (hours) within the EC50 and k**_**1**_**deciles derived from the 5000 parameter sets for the anti-malarial combination therapy, artemether (ART) and lumefantrine (LM).** Top panels are for artemether (EC50 – left hand side, k_max_ right hand side) and bottom panels are for lumefantrine (EC50 – left hand side, k_max_ right hand side).

For artemether-lumefantrine, the proportion of patients cured was correlated with both parameters k_max_ and EC50 of lumefantrine and marginally sensitive to both parameter values of artemether (Figure 6). For lumefantrine, *in vivo* EC50 values of less than 235 ng/ml corresponded to 100% cured and values of 1632 ng/ml and above resulted in approximately 10-20% cured. For this combination therapy, artemether is given at 0, 8, 24, 36, 48 and 60 hours, whereas for the above two combination therapies the artemisinin derivative is given only at 0, 24 and 48 hours. The additional doses of artemether may explain why changes in the EC50 and k_max_ values of artemether are associated with the proportion cured for this artemisinin derivative. This was supported by the results of a simulation run on the first 500 of the 5,000 LHS parameter sets with artemether given at 0, 24 and 48 hours and lumefantrine given at 0, 8, 24, 36, 48 and 60 hours (see Additional file 6). For PCTs, as with the other partner drugs, no associations were observed for the EC50 and k_max_ of lumefantrine (Figure 7). A slight gradient was observed such that longer PCTs were observed for those with higher artemether EC50 values. The values of the slope of either the artemether or lumefantrine concentration-effect curves had no associations with the model outputs (see Additional files 7A-B).

**Figure 8:**
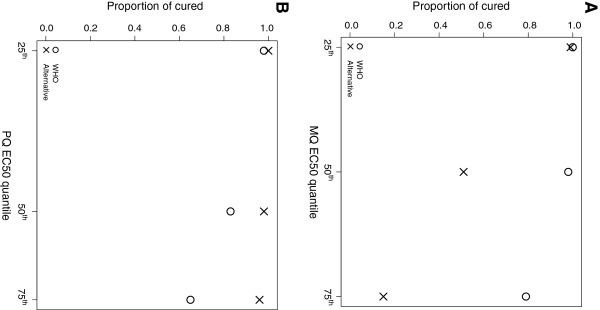
**A-B Proportion of 100 hypothetical patients cured.** Panel **A**: EC50 values of mefloquine (287, 554 and 821 ng/ml) for two different dosing schemes: 8.3 mg/kg at 0, 24 and 48 hours (WHO standard; circles); and 15 mg/kg at 48 hours (alternative scheme; crosses). Panel **B**: EC50 values of piperaquine (32, 53 and 74 ng/ml) for two different dosing schemes: 18 mg/kg at 0, 24 and 48 hours (WHO standard; circles); and 36 mg/kg at 0, 24 and 48 hours (alternative scheme; crosses).

### Comparing alternative dosing regimens

The utility of the mechanistic within host PK-PD model as a decision tool for comparing dosing regimens was investigated for different dosing schemes for each of the partner drugs, mefloquine (WHO recommended dosing of 8.3 mg/kg at 0, 24 and 48 hours *versus* dosing of 15 mg/kg at 48 hours) and piperaquine (WHO recommended dosing of 18 mg/kg at 0, 24 and 48 hours *versus* 36 mg/kg at 0, 24 and 48 hours), administered with the artemisinin derivatives, artesunate and dihydroartemisinin respectively. Since the proportion of patients cured was highly dependent on the EC50 value of the partner drug (a parameter for which we don’t know the value), different dosing schemes were compared for the 25^th^, 50^th^ and 75^th^ percentile values of the 5000 parameter values selected using LHS. All other parameters were fixed at their mean/mode value given in Table 4.

The proportion cured for 100 hypothetical patients with varying PK profiles for the two dosing schemes of mefloquine was 100% when the EC50 value of mefloquine was 287 ng/ml, however, under-dosing patients with only 15 mg/kg of mefloquine resulted in only 50% of the patients being cured if the EC50 is 554 ng/ml and 20% if the EC50 was 821 ng/ml. For the standard recommended mefloquine dose of 8.3 mg/kg at 0, 24 and 48 hours, approximately 95% and 80% would be expected to be cured at the respective EC50 values of 554 and 821 ng/ml (Figure 8A).

Doubling the recommended WHO dose of piperaquine given each day over 3 days results in a higher proportion of patients cured at the EC50 values of 53 and 74 ng/ml for piperaquine (Figure 8B).

## Discussion

The parasitological outcomes simulated in this paper were proportion of patients cured and parasite clearance times, for three different artemisinin-based combination therapies currently recommended by the WHO as the first line treatment for uncomplicated falciparum malaria. This simulation study was comprehensive, randomly drawing from each distribution of the six key pharmacodynamic parameters using Latin-Hypercube-Sampling (LHS), and included between-patient variability in the pharmacokinetic profiles of each anti-malarial drug. The proportion of hypothetical patients cured was observed to be highly correlated to the *in vivo* EC50 and the killing rate (k_max_) of the partner drug co-administered with the artemisinin derivative. However, *in vivo* EC50 values that corresponded to on average 95% of patients cured (a value observed in most clinical efficacy studies of these regimens) were much higher than the values we derived from *in vitro* data (i.e. adjusted *in vitro* IC50), even though the difference in protein binding *in vitro* and *in vivo* were taken into account in the model. *In vitro* experiments typically assess the pharmacodynamic effect of an anti-malarial drug by measuring inhibition of parasite growth in rising concentration of free drug for the length of one parasite life cycle (i.e. 48 hours). The duration of the *in vitro* assay is usually 48 to 72 hours and although this permits a reproducible estimate of parasite drug susceptibility, it may be too short for this estimate to reflect accurately clinical correlates. This may explain in part why *in vitro* measures accord so poorly with the observed pharmacodynamic effect *in vivo*.

There was evidence that the proportion cured decreased if asynchronous infections were treated with artemether-lumefantrine, but the synchronicity of the infection did not strongly influence the proportion cured following treatment with either artesunate-mefloquine or dihydroartemisinin-piperaquine. The PCT for all three artemisinin combination therapies tended to lengthen as the infection became more asynchronous and to shorten as the mean age of the initial parasite burden increased. The finding that asynchronous infections take longer to clear is plausible because it is more likely that there will be parasites outside the killing zone (i.e. early rings and schizonts) during the times when the patient is only exposed to the partner drug (e.g. approximately 7 to 24 hours for mefloquine or piperaquine). The distributions of PCTs in Additional files 4B, Additional file 5B and Additional file 6B are bimodal because the hypothetical patients do not receive a single dose of the artemisinin derivative but are given multiple doses at 24 and 48 hrs (and also 8, 36 and 60 hours for artemether).

The proportion cured following treatment with artesunate-mefloquine was highly correlated to the *in vivo* EC50 value of mefloquine. This is not surprising given the brief time the parasite is exposed to dihydroartemisinin concentrations (approximately 6 hours following each dose of artesunate) compared to mefloquine which remains in the body, on average, for 40 days. This finding concurs with observations from deterministic simulated individual patient parasite versus time profiles using a continuous-time PK-PD model [9]. The association between the proportion cured and the *in vivo* EC50 values of piperaquine and lumefantrine was even stronger than that observed for mefloquine. Both piperaquine and lumefantrine have an enormous volume of distribution and an elimination profile that comprises a steep short distribution phase followed by a slow elimination phase from day 5–7 onwards [19,30]. However, lumefantrine has a much lower volume of distribution compared with piperaquine and this explains why higher values of *in vivo* EC50 for lumefantrine are required before the simulated observations predict, on average, 10-20% cured. PCTs did not increase when the maximal killing rate (k_max_) of artesunate/dihydroartemisinin decreased, although this was observed for the ring stage parasites in the discrete-time PK-PD model reported by Saralamba *et al.*[10]. These conflicting findings are likely to arise for a number of reasons. First, in this paper it was assumed that the maximal killing rate of the artemisinin derivatives was constant across the killing zone (i.e. the age range of parasites for which the drug kills) since stage-specific killing rates for each anti-malarial was not known. Second, k_max_ values for the artemisinin derivatives were randomly selected from a Parasite Reduction Ratio at 48 hours (PRR_48_) ranging from 5 × 10^4.28^ to 5 × 10^6.28^ with a mode value of 10^5.28^[3] parasites reduced every 48 hours. In the observations by Saralamba *et al.*[10], the maximal killing rate of artesunate observed for the ring stages with delayed PCTs was a mean of 62% /cycle corresponding to a much lower PRR (~10^0.42^) than our minimal value. Third, in this study the partner drugs were administered at the same times as the doses of the artemisinin derivatives and therefore contributed to the parasite clearance times whereas in Saralamba *et al.* only artesunate was administered in the first 48 hours of treatment.

This simulation study has a number of strengths which includes: the method of LHS for randomly selecting 5000 sets of the pharmacodynamic parameter values combined with simulations of 100 pharmacokinetic profiles for each anti-malarial to capture between-patient variability in drug exposure. Moreover our comparison of alternative dosing regimens highlight the utility of PK-PD models to compare dosing schemes and have the capacity to examine the association between a range of PK parameters (e.g. time above therapeutic concentration, maximum concentration or area under the concentration-time profile) and parasitological outcome. The limitations of this study were: the within-host PK-PD model assumes that the background immunity of the hypothetical patients was low or absent; no pharmacodynamic synergism between the two anti-malarials of each combination therapy evaluated was assumed; and the a priori assumption that the maximal killing rate of the artemisinin derivatives and the partner drugs remained constant across the different ages of the parasite within the defined killing zone (e.g. 6 to 44 hours for artesunate). Furthermore, the median PCTs were approximately 24 hours whereas in many clinical studies approximately 48 hours is often observed. This difference may be due to one or a combination of factors including: an assumption that there was no synergy between the drugs; the maximal killing rate of the partner drug, piperaquine, was taken from an *in vitro* experiment and thus may be higher than observed *in vivo*; and the number of circulating parasites was calculated in time steps of one hour post initial treatment for determining PCT whereas in clinical efficacy studies this is often determined from blood smears collected only every 24 hours.

In conclusion, this simulation study demonstrates the utility of using mechanistic within-patient PK-PD models for comparing parasitological outcomes of different dosing schemes of anti-malarial treatments and different anti-malarial combination therapies. The findings of this study suggest that the parasitological outcomes be compared for a number of scenarios of the pharmacodynamic parameter values, especially the unknown *in vivo* EC50 value. These simulation studies should not be used as a replacement to conducting the clinical efficacy trials but instead used to assist in determining the best dosing schemes and potential partner drugs to be considered for new anti-malarial treatments. This simulation-based approach has the potential to reduce the number of clinical efficacy trials carried out in the Phase II and Phase III stages of drug development, which will reduce the cost of drug development, speed up the process of drug registration, and could help identify non-ethical trials of malaria patients.

## Competing interests

The authors declare that they have no competing interests.

## Authors' contributions

JAS developed the idea for a simulation study with AH, SC, RP, JM, JG-B, JMcC and SZ making contributions to the concept and design of the study. SC, AH and RP were involved in the acquisition of data required to obtain estimates of (or ranges for) the model parameters. SZ wrote the R code to run the simulation-based decision tool with contributions from JMcC, KS and KJ. JAS and SZ wrote the first draft of the paper and together with AH, SC, RP, JM, JG-B, JMcC, KJ and KS contributed to the interpretation of the simulated output. All authors reviewed the paper and approved the final version.

## Supplementary Material

Additional file 1 **Age distribution of initial parasites burden.** Simulated number of parasites (/μL of blood) at each stage of the life cycle – for a patient with a pre-treatment parasite burden of 10^11^ parasites, a mean parasite age of 8 hours and a standard deviation of 12 hours. Click here for file

Additional file 2 **Simulated pharmacokinetic profiles.** Simulated pharmacokinetic profiles of dihydroartemisinin, artemether, mefloquine, lumefantrine and piperaquine for the 100 hypothetical patients used by the Latin hypercube sampling (LHS). Superimposed on the profiles (in a different colour) is the mean population PK profile. Click here for file

Additional file 3 **Tornado plots.** Tornado plots of partial rank correlation coefficients, indicating the importance of each drug dependent parameter’s (EC50, k_max_ and γ) uncertainty in contributing to the variability in the proportion cured (left) and parasite clearance time (PCT) (right) for each artemisinin combination therapy. Click here for file

Additional file 4 **A-B: Proportion cured and parasite clearance time (PCT) for 100 hypothetical patients treated with artesunate (ARS) and mefloquine (MQ) combination therapy.** Proportion cured and PCT were calculated for each set of Latin hypercube sampled (LHS) pharmacodynamic parameter values over 100 hypothetical patients with varying ARS and MQ pharmacokinetic profiles. Panel A: Pharmacodynamic parameters sampled using LHS versus proportion cured. Panel B: Pharmacodynamic parameters sampled using LHS versus PCT. Click here for file

Additional file 5 **A-B: Proportion cured and parasite clearance time (PCT) for 100 hypothetical patients treated with dihydroartemisinin (DHA) and piperaquine (PQ) combination therapy.** Proportion cured and PCT were calculated for each set of Latin hypercube sampled (LHS) pharmacodynamic parameter values over 100 hypothetical patients with varying DHA and PQ pharmacokinetic profiles. Panel A: Pharmacodynamic parameters sampled using LHS versus proportion cured. Panel B: Pharmacodynamic parameters sampled using LHS versus PCT. Click here for file

Additional file 6 **Distribution of proportion cured for a simplified artemether-lumefantrine dosing regimen.** Distribution of proportion cured within the EC50 and k_max_ deciles derived from the first 500 of the 5000 parameter sets for the antimalarial combination therapy, artemether (ART) and lumefantrine (LM) where artemether was given at 0, 24 and 48 hours and lumefantrine was given at 0, 8, 24, 36, 48 and 60 hours. Top panels are for artemether (EC50 – left hand side, k_max_ right hand side) and bottom panels are for lumefantrine (EC50 – left hand side, k_max_ right hand side). Click here for file

Additional file 7 **A-B: Proportion cured and parasite clearance time (PCT) for 100 hypothetical patients treated with artemether (ART) and lumefantrine (LF) combination therapy.** Proportion cured and PCT were calculated for each set of Latin hypercube sampled (LHS) pharmacodynamic parameter values over 100 hypothetical patients with varying ART and lumefantrine LF pharmacokinetic profiles. Panel A: Pharmacodynamic parameters sampled using LHS versus proportion cured. Panel B: Pharmacodynamic parameters sampled using LHS versus PCT. Click here for file
