# Supplementary figures and images for: Assessing the utility of an anti-malarial pharmacokinetic-pharmacodynamic model for aiding drug clinical development
Source: Malar J. 2012 Aug 30;11:303. doi: 10.1186/1475-2875-11-303 (PMC3546862; doi:10.1186/1475-2875-11-303)

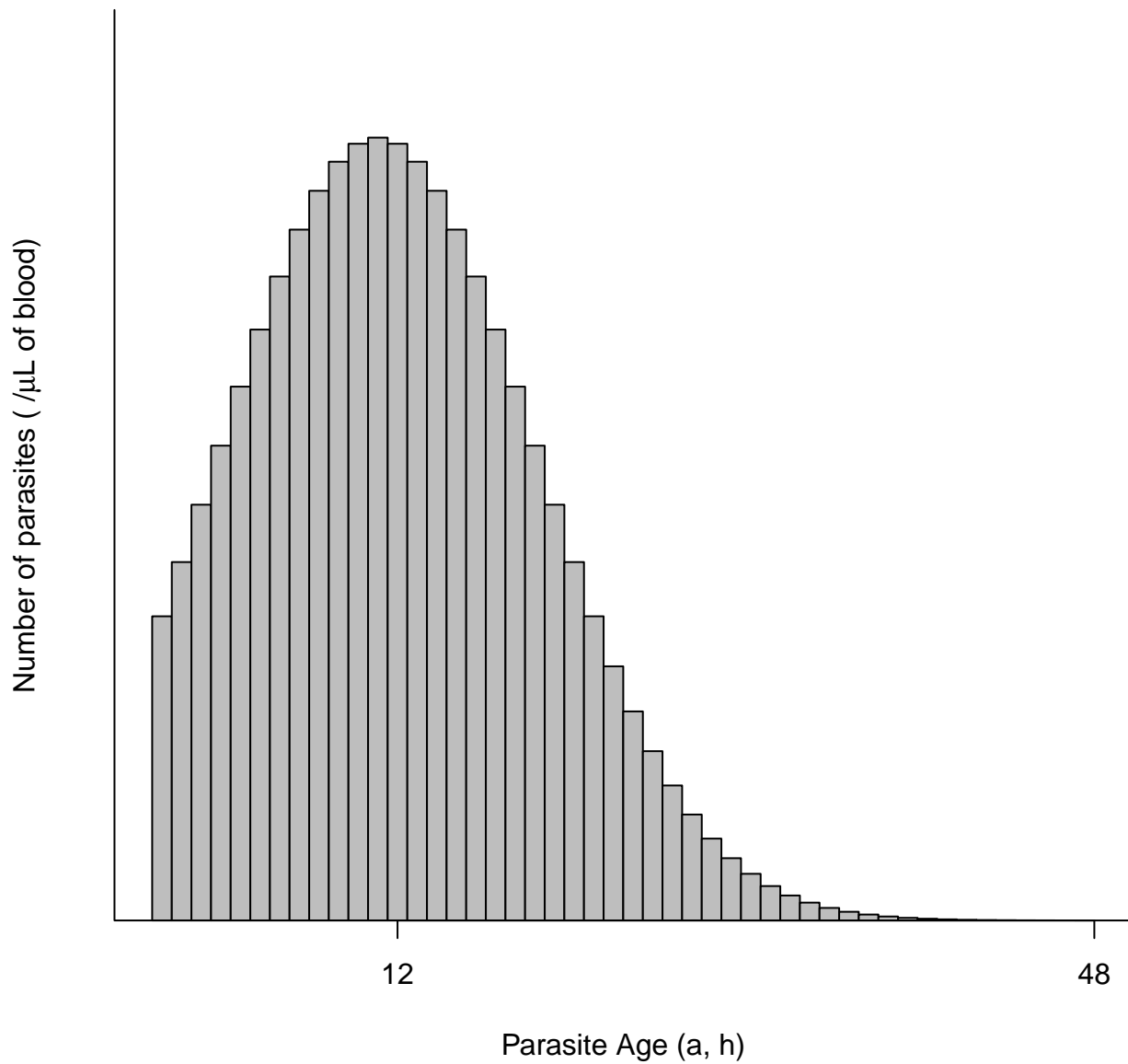

Supplement: Additional file 1 — Age distribution of initial parasites burden. Simulated number of parasites (/μL of blood) at each stage of the life cycle – for a patient with a pre-treatment parasite burden of 1011 parasites, a mean parasite age of 8 hours and a standard deviation of 12 hours. [file 1475-2875-11-303-S1.pdf]

**DHA**

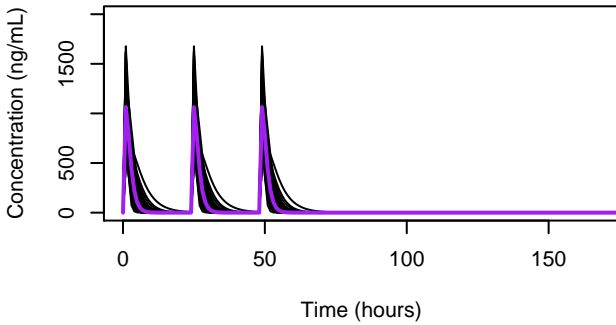

**Mefloquine**

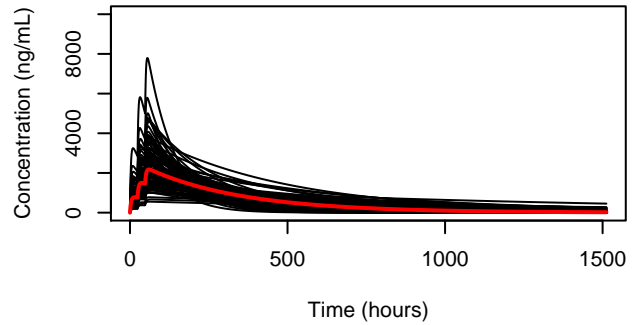

**Artemether**

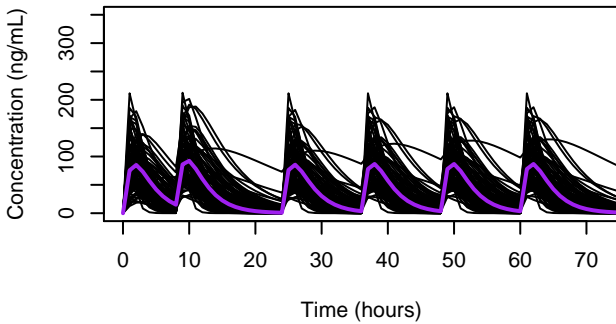

**Lumefantrine**

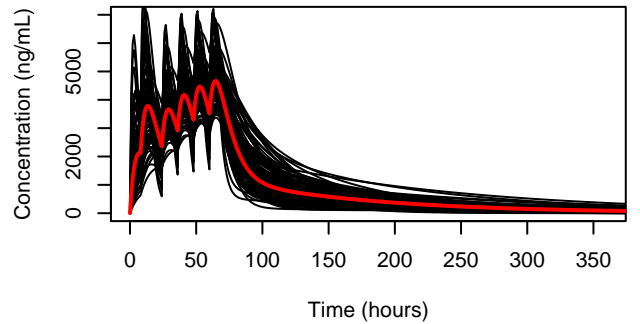

**Piperaquine**

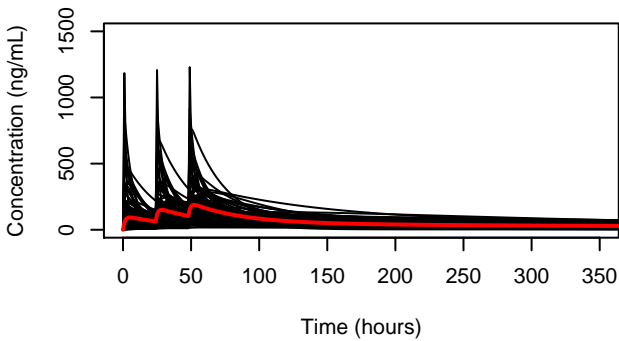

Supplement: Additional file 2 — Simulated pharmacokinetic profiles. Simulated pharmacokinetic profiles of dihydroartemisinin, artemether, mefloquine, lumefantrine and piperaquine for the 100 hypothetical patients used by the Latin hypercube sampling (LHS). Superimposed on the profiles (in a different colour) is the mean population PK profile. [file 1475-2875-11-303-S2.pdf]

ARS+MQ

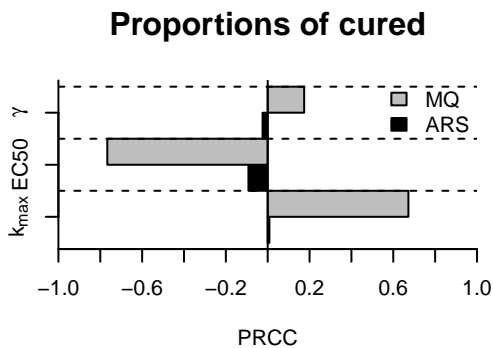

PCTs

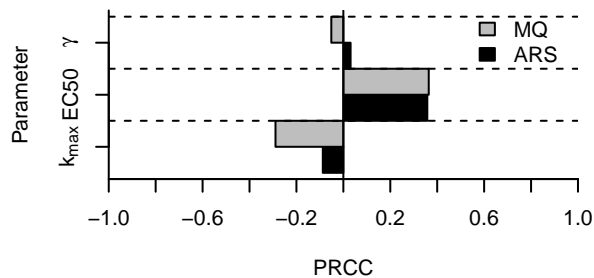

DHA+PQ

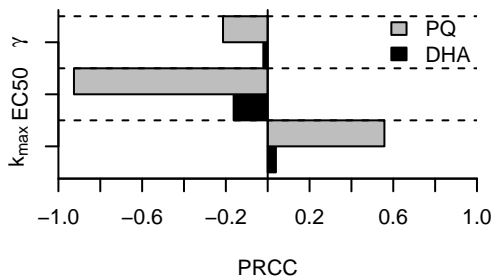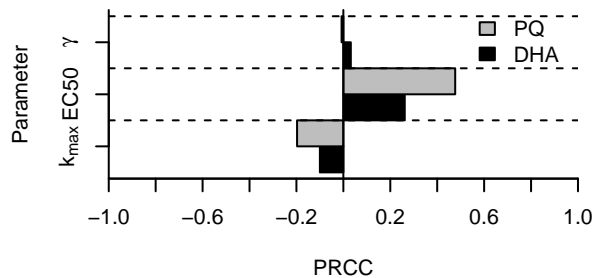

ART+LF

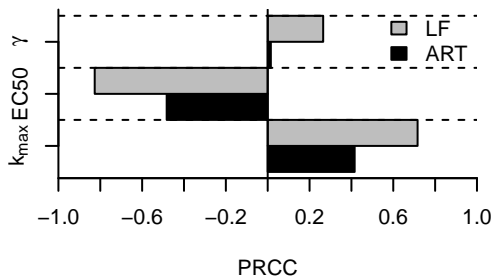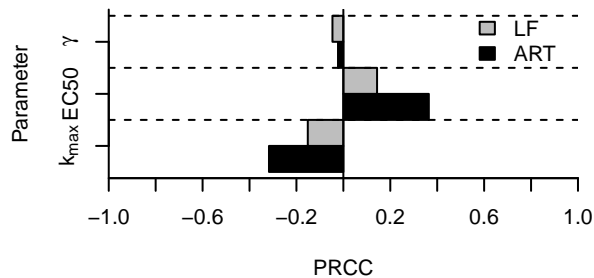

Supplement: Additional file 3 — Tornado plots. Tornado plots of partial rank correlation coefficients, indicating the importance of each drug dependent parameter’s (EC50, kmax and γ) uncertainty in contributing to the variability in the proportion cured (left) and parasite clearance time (PCT) (right) for each artemisinin combination therapy. [file 1475-2875-11-303-S3.pdf]

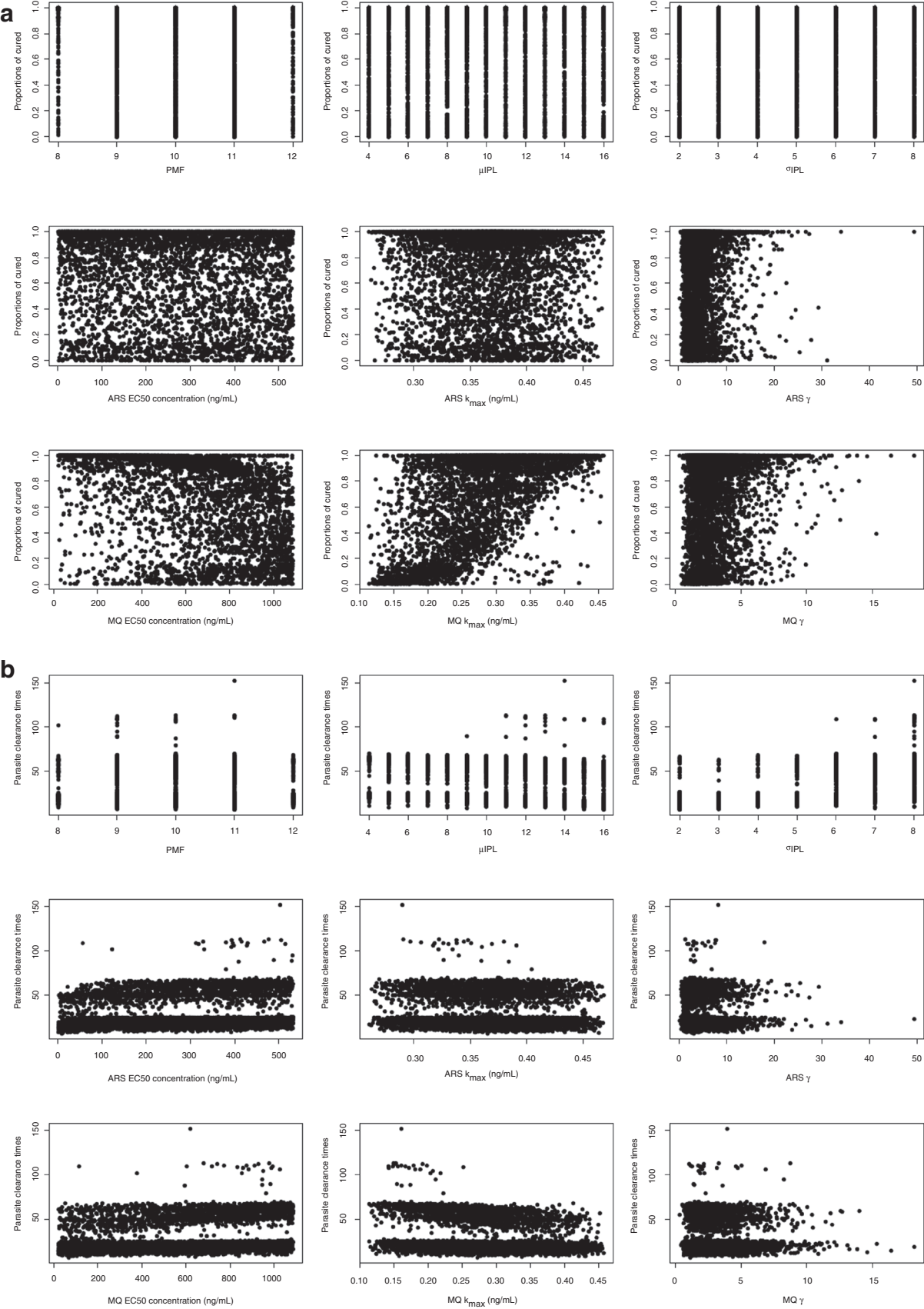

Supplement: Additional file 4 — A-B: Proportion cured and parasite clearance time (PCT) for 100 hypothetical patients treated with artesunate (ARS) and mefloquine (MQ) combination therapy. Proportion cured and PCT were calculated for each set of Latin hypercube sampled (LHS) pharmacodynamic parameter values over 100 hypothetical patients with varying ARS and MQ pharmacokinetic profiles. Panel A: Pharmacodynamic parameters sampled using LHS versus proportion cured. Panel B: Pharmacodynamic parameters sampled using LHS versus PCT. [file 1475-2875-11-303-S4.pdf]

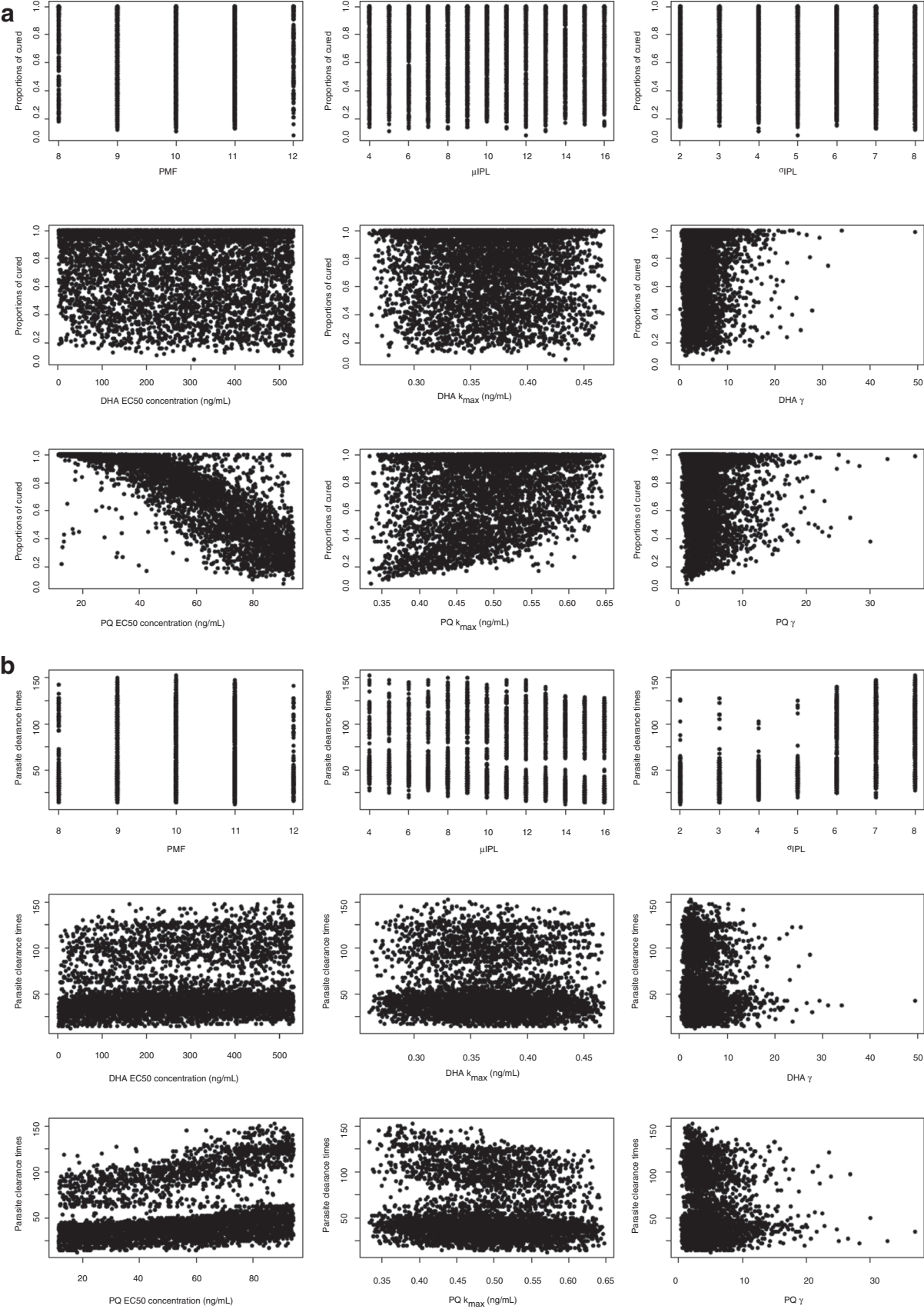

Supplement: Additional file 5 — A-B: Proportion cured and parasite clearance time (PCT) for 100 hypothetical patients treated with dihydroartemisinin (DHA) and piperaquine (PQ) combination therapy. Proportion cured and PCT were calculated for each set of Latin hypercube sampled (LHS) pharmacodynamic parameter values over 100 hypothetical patients with varying DHA and PQ pharmacokinetic profiles. Panel A: Pharmacodynamic parameters sampled using LHS versus proportion cured. Panel B: Pharmacodynamic parameters sampled using LHS versus PCT. [file 1475-2875-11-303-S5.pdf]

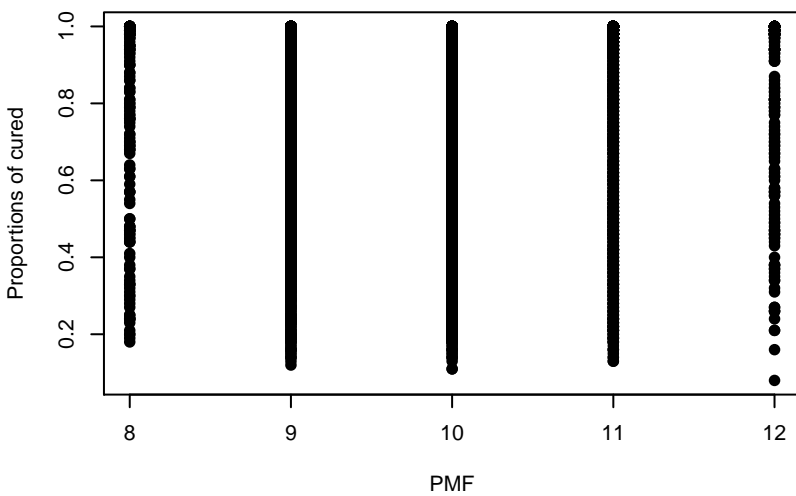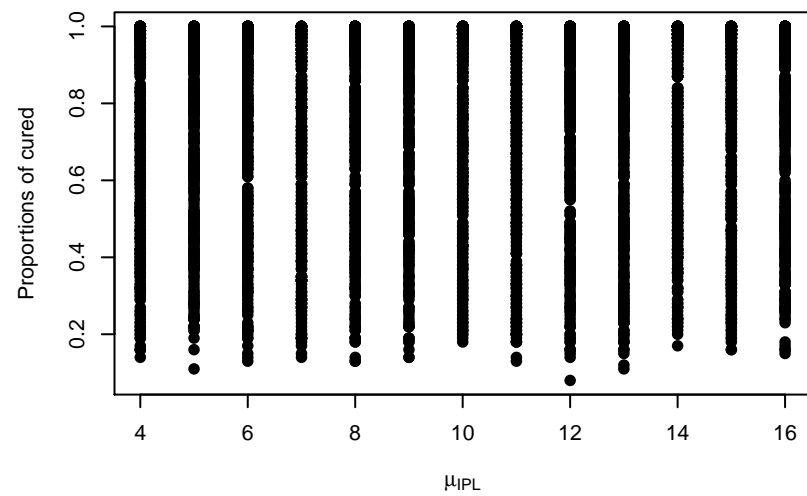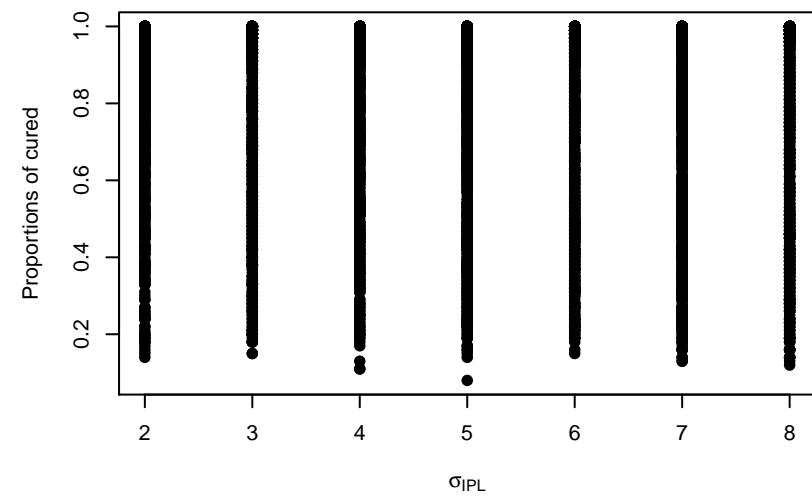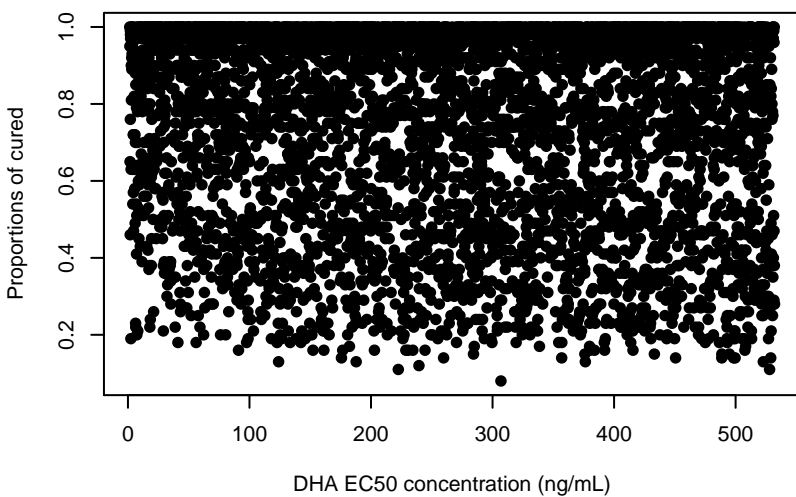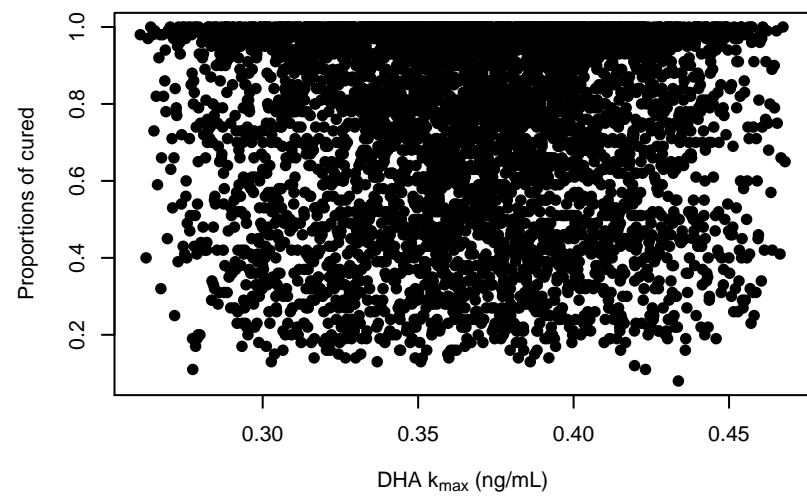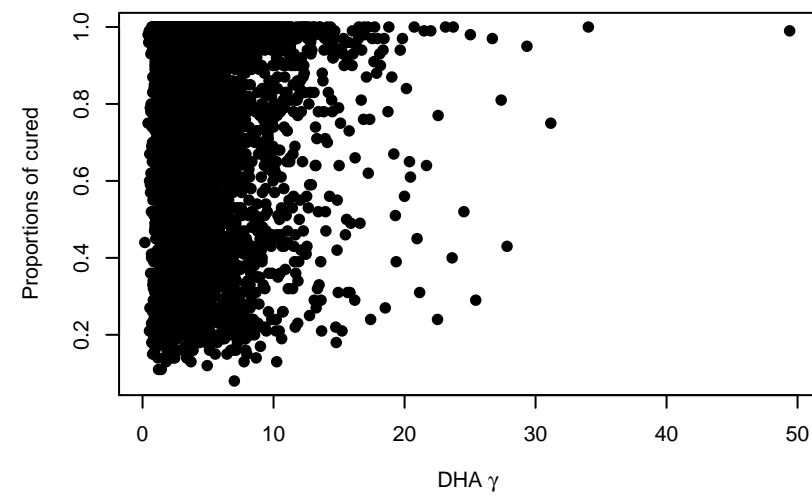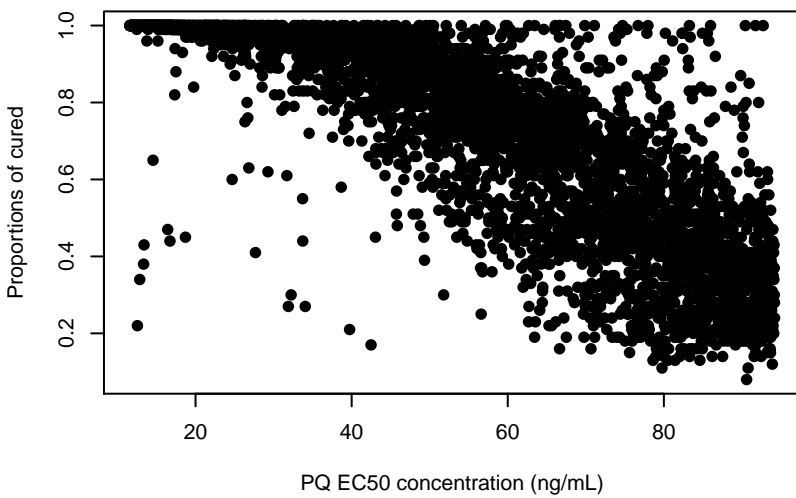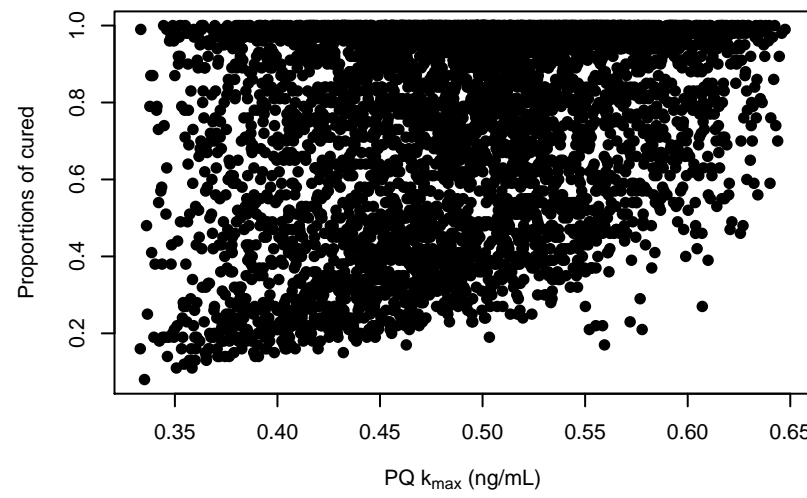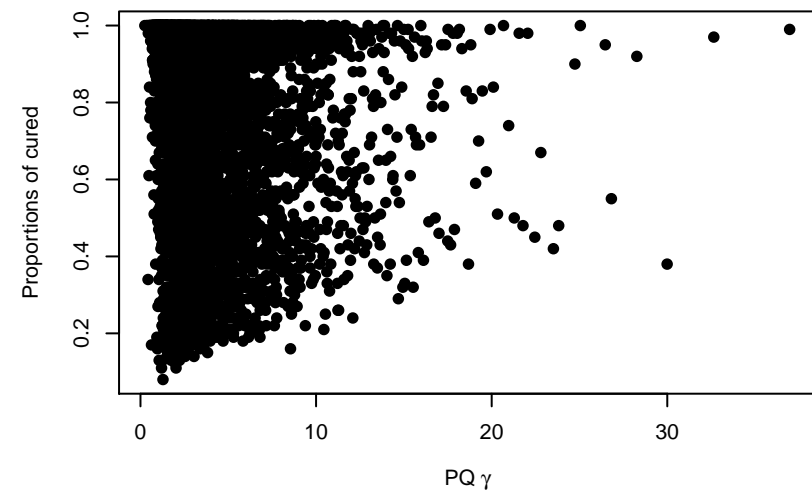

Supplement: Additional file 6 — Distribution of proportion cured for a simplified artemether-lumefantrine dosing regimen. Distribution of proportion cured within the EC50 and kmax deciles derived from the first 500 of the 5000 parameter sets for the antimalarial combination therapy, artemether (ART) and lumefantrine (LM) where artemether was given at 0, 24 and 48 hours and lumefantrine was given at 0, 8, 24, 36, 48 and 60 hours. Top panels are for artemether (EC50 – left hand side, kmax right hand side) and bottom panels are for lumefantrine (EC50 – left hand side, kmax right hand side). [file 1475-2875-11-303-S6.pdf]

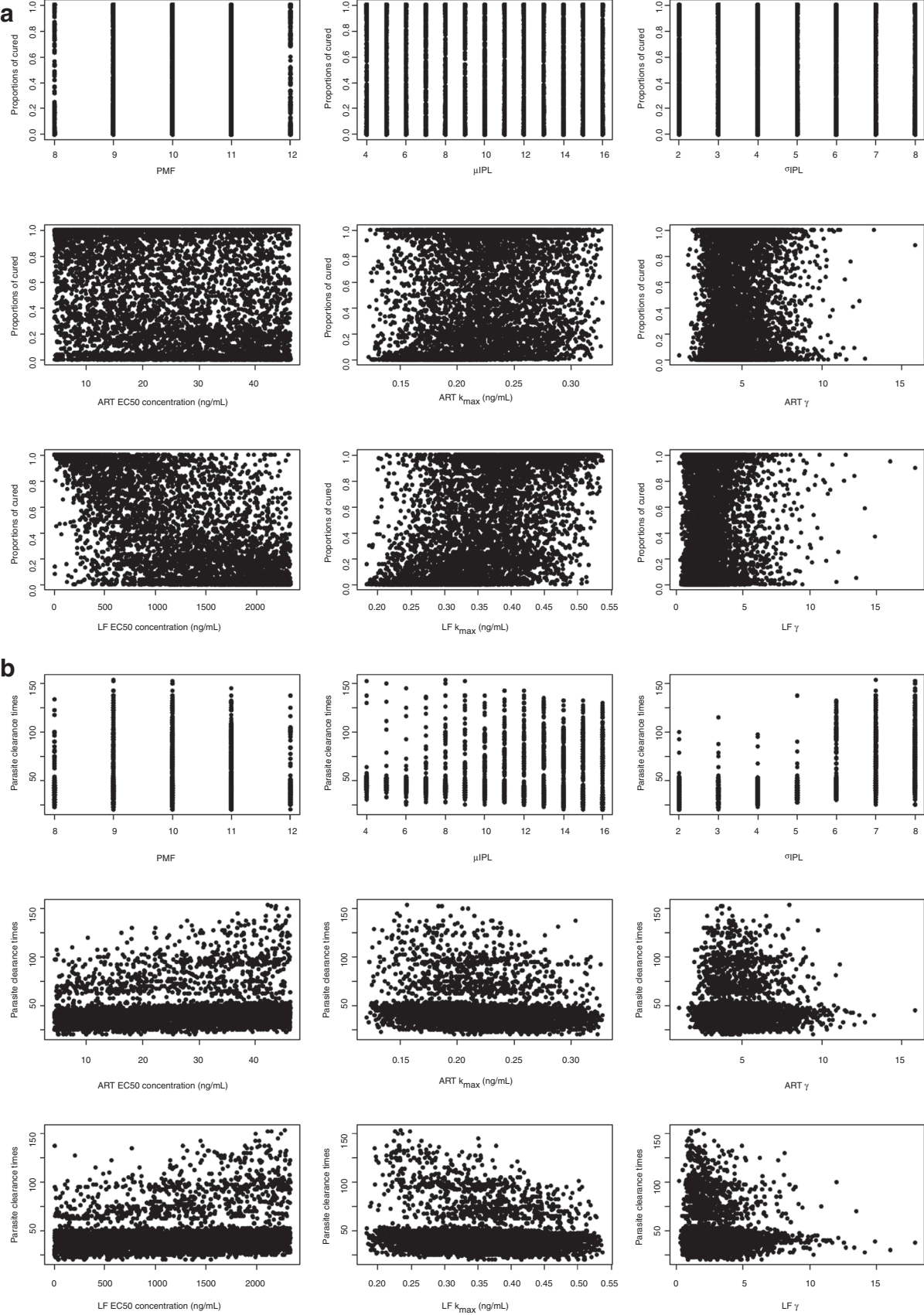

Supplement: Additional file 7 — A-B: Proportion cured and parasite clearance time (PCT) for 100 hypothetical patients treated with artemether (ART) and lumefantrine (LF) combination therapy. Proportion cured and PCT were calculated for each set of Latin hypercube sampled (LHS) pharmacodynamic parameter values over 100 hypothetical patients with varying ART and lumefantrine LF pharmacokinetic profiles. Panel A: Pharmacodynamic parameters sampled using LHS versus proportion cured. Panel B: Pharmacodynamic parameters sampled using LHS versus PCT. [file 1475-2875-11-303-S7.pdf]
